# Impact of Inherited Prothrombotic Disorders on the Long-Term Clinical Outcome of Percutaneous Transluminal Angioplasty in Patients with Diabetes

**DOI:** 10.1155/2015/369758

**Published:** 2015-07-13

**Authors:** Michal Dubský, Alexandra Jirkovská, Libuše Pagáčová, Robert Bém, Andrea Němcová, Vladimíra Fejfarová, Veronika Wosková, Edward B. Jude

**Affiliations:** ^1^Diabetes Centre, Institute for Clinical and Experimental Medicine, 140 21 Prague, Czech Republic; ^2^Diabetes Centre, Tameside Hospital NHS Foundation Trust and University of Manchester, Ashton-Under-Lyne OL6 9RW, UK

## Abstract

The aim of our study was to analyse inherited thrombotic disorders that influence the long-term outcome of PTA. *Methods*. Diabetic patients with peripheral arterial disease (PAD) treated by PTA in our centre between 2008 and 2011 were included in the study. Patients were divided into unsuccessful PTA group (75 patients), successful PTA group (58 patients), and control group (65 patients, with diabetes but no PAD). Diagnosis of inherited thrombotic disorders included mutation in factor V (Leiden), factor II (prothrombin), and mutation in genes for methylenetetrahydrofolate reductase—MTHFR (C677T and A1298C). *Results*. The genotypic frequency of Leiden allele G1691A was significantly associated with a risk of unsuccessful PTA in comparison with successful PTA group and control group (OR 8.8 (1.1–70.6), *p* = 0.041, and OR 9.8 (1.2–79.2), *p* = 0.032, resp.). However, we only observed a trend for the association of the prothrombin allele G20210A and risk of PTA failure. The frequencies of alleles of MTHFR 677 or 1298 did not differ significantly among the groups. *Conclusion*. Our study showed higher frequency of heterozygous form of Leiden mutation in diabetic patients with unsuccessful outcome of PTA in comparison with patients with successful PTA and diabetic patients without PAD.

## 1. Introduction

Thrombophilia is defined as a hypercoagulable state that predisposes to arterial or venous thrombosis. The association between inherited prothrombotic disorders and venous thrombosis has been demonstrated in many large studies [1, 2]; on the other hand their relation to arterial thrombosis is described only in case reports or few small studies [3–5].

Thrombosis could be one of the important factors that influence the clinical outcome of percutaneous transluminal angioplasty (PTA) [6]. The immediate effect of PTA is influenced mainly by the number of patent arteries [7], whereas the long-term clinical outcome can be modified by possible later restenosis (thrombosis or intimal proliferation) even in patients after stenting [8]. Several studies have investigated the prevalence of inherited thrombophilia in peripheral arterial disease (PAD). Reny et al. studied the association between G20210A prothrombin and FVArg506Gln polymorphisms and PAD in a case-control study [9]. Another study assessed the presence of thrombophilic alterations and cardiovascular events in patients who underwent endovascular intervention for PAD [10].

Diabetic patients are usually affected by different abnormalities of the coagulation cascade and may be predisposed to thrombosis mostly because of metabolic changes and acquired or inherited coagulation defects [11].

Coagulation is a highly regulated process dependent on appropriate aggregation of platelets and activation of circulating and tissue-bound coagulation factors [12]. One of the important acquired coagulation disorders in patients with diabetes is impaired fibrinolysis. It has been shown that levels of complement C3 were associated with thinner fibrin fibers and prolonged time of clot lysis in diabetic patients [13].

Inherited prothrombotic disorders may include deficiencies of anticoagulants such as protein C, protein S or antithrombin III, or specific mutations of factor II—prothrombin (G20210A)—or factor V (FV) Leiden that account for 25% of inherited prothrombotic states [14].

A single point mutation in the factor V Leiden (G1691A) that specifically converts amino acid at 3 sites (most importantly arginine 506 to glutamine) leads to an inability of APC to cleave factor V (the mutant is strongly resistant to APC) and thus to a hypercoagulable state [15]. The prevalence of the heterozygous form of Leiden mutation is seen in 3–5% of the general population and increases the risk of thromboembolism sevenfold to tenfold [15, 16].

A specific point mutation in the prothrombin gene (conversion of glutamine to arginine at nucleotide 20210 (G20210A)) leads to an increase in plasma levels of prothrombin [17]. The prevalence of heterozygous form of this mutation in the general population is about 1-2% and is associated with a two- to fourfold increased risk of thromboembolism [16].

Increased plasma levels of homocysteine have also been proven to be one of the key factors in the development of venous or arterial thrombosis [18], and plasma levels can be influenced by avitaminoses of vitamins B6, B12, and folate. Coagulation defects are also strongly associated with mutation in methylenetetrahydrofolate reductase (MTHFR) at 2 positions of nucleotides—C677T and A1298C [5, 19]. The mutation in MTHFR leads to defect in transformation of homocysteine into methionine and to increase in serum levels of homocysteine, which enhances platelet activation, thromboxane production, and platelet aggregation [20].

The published data about the incidence of inherited prothrombotic disorders in diabetic patients is lacking. Diabetic patients after unsuccessful PTA are considered as a risk group for development of thrombosis and therefore possibly a major amputation.

The aim of our study was to analyze selected inherited thrombophilia factors that influence the long-term outcome of PTA and to compare them with diabetic patients without peripheral arterial disease (PAD).

## 2. Methods

Diabetic patients with chronic critical limb ischemia treated by PTA in our centre between January 2008 and December 2011 were consecutively included into this controlled study. Critical limb ischaemia (CLI) was defined in accordance with TASC II [21] as ulcers or gangrene attributable to objectively proven arterial occlusive disease. All patients had PEDIS stage 3 with TcPO_2_ < 30 mm Hg or ankle brachial index < 0.6 [22] and Rutherford category 5-6 [23].

The study was designed as cross-sectional. Patients were divided into 3 groups. First group included 75 patients after unsuccessful PTA which was defined as a necessity for re-PTA or bypass or TcPO_2_ below 40 mm Hg at one-year follow-up after the initial revascularization. Fifty-eight diabetic patients were enrolled into the successful PTA group. Diabetic controls were selected from a database of our hospital. All diabetic patients, who were consecutively examined for genetics of thrombophilia during the same period as patients who underwent the PTA (January 2008–December 2011) and were free of PAD according to hospital notes, were enrolled into the control group (65 patients). The reasons for the thrombophilia examination were mainly ischemic heart disease (atrial fibrilation and status after myocardial infarction—28%), status after stroke (21%), deep venous thrombosis in history (16%), status after organ transplant (kidney, pancreas, and liver—14%), examination before organ transplant (10%), gynecological abnormalities (8%), and vasculitis (3%).

The study was approved by the local ethics committee and all patients signed informed consent. Main demographic and other characteristics of patients among all groups are shown in Table 1.

There was no significant difference in patient characteristics between groups at baseline, including age, gender, glycated hemoglobin, duration of diabetes, and presence of comorbidities (Table 1). Patients in both PTA groups did not significantly differ in usage of dual antiplatelets (25.3 versus 29.3%) or anticoagulant therapy (10.7 versus 8.6%).

Inherited thrombophilia was assessed by heterozygous form of mutation of factor V Leiden and factor II prothrombin and mutations of both heterozygous and homozygous forms of MTHFR (positions of nucleotides C677T and A1298C). Patients in all groups also did not significantly differ in blood levels of other coagulation parameters (protein C and S, antithrombin III, fibrinogen, D-dimers, and homocysteine; Table 2). All samples were taken from peripheral blood before the PTA and homozygous and heterozygous forms of each mutation were analyzed. DNA was isolated from whole blood samples using the NucleoSpin Blood Kit (Macherey-Nagel, Düren, Germany). DNA was isolated and analyzed according to the manufacturer's instructions. The thrombophilic mutations were detected using the thrombophilic kits (GeneProof, Brno, Czech Republic) designed for the detection of mutation in the genes for prothrombin (factor II), factor V Leiden, MTHFR A1298C, and MTHFR C677T by the real-time PCR method. This method is based on the PCR amplification and on the hybridization of the amplified sequence with fluoroform marked probes for the standard allele and for the mutant allele of the above-mentioned genes. Evaluation included standard homozygote, mutant heterozygote, and mutant homozygote.

All data were expressed as mean ± standard deviation. The statistical significance was analyzed using the Hardy-Weinberg equilibrium test based on the chi-square goodness-of-fit test and odds ratio associated with a thrombophilic abnormality was calculated. Chi-square test and Fisher's test of combination of several mutations were analyzed.

## 3. Results

The genotypic frequencies of Leiden mutation (G1691A), prothrombin mutation (G20210A), and MTHFR C677T and A1298C mutations were in Hardy-Weinberg equilibrium (Table 3). The Leiden allele G1691A was significantly associated with a risk of unsuccessful PTA with an OR of 8.8 (1.1–70.6), *p* = 0.041, in comparison with successful PTA group and with an OR 9.8 (1.2–79.2), *p* = 0.032, in comparison with control group. On the other hand, the prothrombin allele G20210A was not significantly associated with a risk of PTA failure—with an OR 3.8 (0.8–18.4), *p* = 0.095, in comparison with successful PTA group and with an OR 4.3 (0.9–20.7), *p* = 0.069, in comparison with control group; we observed only a trend for the association. The frequencies of alleles of MTHFR 677 or 1298 did not differ significantly among the groups (Table 3).

The combination of heterozygous form of MTHFR and Leiden mutations was significantly more prevalent in the unsuccessful PTA group compared to successful PTA group and control group (22.4 versus 8 versus 13.8%, *p* = 0.008), but combination of MTHFR and prothrombin did not differ significantly. The analysis of combination of MTHFR and elevated homocysteine did not significantly increase the risk of thrombosis after PTA. We did not observe an association in the homozygous form of mutations of Leiden or prothrombin amongst groups.

## 4. Discussion

The results of our study suggest that inherited thrombophilia can be more frequent in patients after unsuccessful PTA. The only factor significantly associated with an unsuccessful PTA was factor V Leiden mutation; factor II prothrombin mutation was not associated with unsuccessful PTA, but only a trend was observed. The combination of MTHFR and factor V Leiden mutations was also associated with increased risk of restenosis.

Diabetic patients with successful PTA and diabetic patients without PAD had the frequency of inherited thrombophilia similar to the general population (1–4%) which is in accordance with published data [15, 16].

A restenosis after PTA is a major complication that compromises the long-term clinical outcome of the endovascular procedure [24, 25]. Inherited prothrombotic disorders are only one of the mechanisms responsible for development of restenosis. Injury and repair of the vascular wall after PTA is influenced by multiple factors that could initiate a restenosis after PTA. One factor is a mural thrombosis, which may be dependent on platelet aggregation; the other mechanisms are proliferation of smooth muscle cells and increased inflammatory response. Direct injuries during angioplasty (dissection, intimal laceration, or plaque compression) may lead to platelet activation and release of growth factors which mediate endothelial cells, smooth muscle cells, and increase in extracellular matrix, all of which may result in the narrowing of the vascular lumen [26]. Zuojun et al. have shown that inducible protein- (IP-) 10 (member of the CXC chemokine family), which is overexpressed in diabetic patients (29), may play an important role in intimal hyperplasia because of its chemotactic effects and induction of smooth muscle cell proliferation [24, 27].

Studies about the association between arterial thrombosis and thrombophilia are lacking, but some studies have described the association between venous thrombosis and inherited thrombophilia. Tepe et al. observed in a meta-analysis of more than 11000 patients with recurrent venous thrombosis a strong evidence for hyperhomocysteinaemia, factor V mutation, and high D-dimer, fibrinogen, and factors VIII and IX as risk factors for recurrence of venous thromboembolism [6]. Gemmati et al. analyzed the interaction between MTHFR homozygotes and prevalence of arterial and venous thrombosis and demonstrated that the homozygosity for MTHFR mutation increases the risk for both arterial and venous thromboses [28].

Sartori et al. described the role of inherited thrombophilia for cardiovascular risk in 230 patients after PTA [10]. The composite endpoint—major adverse cardiovascular events (which included all-cause mortality, myocardial infarction, hospitalisation for unstable angina, stroke, percutaneous coronary intervention, coronary artery bypass surgery, and lower extremity and carotid surgery)—was reached in 41% of patients during mean follow-up of 24 months. Single inherited prothrombotic mutations were not the predictors of those events, whereas patients with two or more thrombophilic alterations had a higher risk of cardiovascular events than those with one or no thrombophilic abnormalities. The endpoint of this study was also different from our study: Sartori and colleagues did not follow up for restenosis after PTA and the study prospectively followed the occurrence of cardiovascular events during a 2-year follow-up. In contrast with this paper we did not find any difference in the presence of chronic ischemic heart disease, hypertension, and stroke at baseline among the study groups. We also observed a significant influence of combination of inherited prothrombotic disorders on the restenosis after PTA similarly as Sartori et al. observed a similar influence on cardiovascular events.

Reny et al. demonstrated that prothrombin allele G20210A polymorphism was more frequently present in PAD patients after adjustment for diabetes, smoking, hypertension, and dyslipidemia [9]; although the Leiden allele FVArg506Gln was not significantly associated with PAD in this study, those findings are in contrast with our results. On the other hand Sampran proved that Leiden mutation was significantly increased in patients with PAD [29].

The two main limitations of our study were the cross-sectional design and small sample size; therefore we consider results from our study as preliminary.

## 5. Conclusions

In conclusion, our study showed significantly higher frequency of heterozygous form of Leiden mutation in diabetic patients with unsuccessful clinical outcome of PTA during one-year follow-up, in comparison with patients after successful PTA and diabetic patients without PAD. A high prevalence of thrombophilia in the group of patients after unsuccessful PTA (more than 10%) suggests the need for more frequent examinations of these mutations in patients with diabetes and CLI after complicated PTA and possibly an adequate antithrombotic or anticoagulation therapy. Larger prospective studies are needed to investigate the benefit of the latter therapies in this high risk group of diabetic patients.

## Figures and Tables

**Table 1:** Patients' baseline characteristics^*∗*^.

Parameter	Unsuccessful PTA group (*n* = 75)	Successful PTA group (*n* = 58)	Control group (*n* = 65)
Age (years)	64.6 ± 10.3	63.5 ± 9.2	58.4 ± 14.2
Gender (% of men)	74.2	73.3	68.7
Diabetes duration (years)	21.6 ± 10.9	24.5 ± 14	20.6 ± 9.3
HbA_1_c (mmol/mol)	62.5 ± 12.2	64.6 ± 17.3	59.8 ± 15.6
Ischemic heart disease (%)	65.3	62.1	58.4
Stroke (%)	12.7	13	15.3
Chronic renal insufficiency CKD 2–4 (%)	29.3	25.8	27.7
Hypertension (%)	74.1	76	78.4
Mean total cholesterol	4.2 ± 0.9	4.3 ± 0.8	4.6 ± 1.1
Mean LDL cholesterol	2.5 ± 0.7	2.4 ± 0.7	2.7 ± 0.9
Active smoking (%)	34.7	29.3	30.7

^*∗*^No significant difference between groups.

**Table 2:** Mean values of coagulation factors among the groups^*∗*^.

Parameter	Unsuccessful PTA group (*n* = 75)	Successful PTA group (*n* = 58)	Control group (*n* = 65)
Protein C (mg/L)	115.3 ± 32.3	104.8 ± 33.6	102.4 ± 45.4
Protein S (mg/L)	98.6 ± 31.4	94.1 ± 37.6	90.2 ± 33.7
Antithrombin III (%)	98.8 ± 16	98.8 ± 16.6	96.9 ± 20.2
Fibrinogen (g/L)	4.9 ± 1.3	5.2 ± 1.4	4.8 ± 1.3
D-dimers (mg/L)	1.0 ± 0.8	0.8 ± 0.5	0.9 ± 0.8
Homocysteine (*μ*mol/L)	17.7 ± 12.5	14.7 ± 8.5	14.8 ± 5.8

^*∗*^No significant difference between groups.

**Table 3:** Genotype frequencies with odds ratios.

Polymorphism	Genotype	Unsuccessful PTA (*n* = 75)	Successful PTA (*n* = 58)	Control (*n* = 65)	OR unsuccessful versus successful PTA groups	OR unsuccessful PTA versus control group
	GG	65 (86.7%)	57 (98.3%)	63 (96.9%)		
Leiden	GA	10 (13.3%)	1 (1.7%)	2 (3.1%)	8.8 (1.1–70.6)	9.8 (1.2–79.2)
G1691A	AA	0 (0%)	0 (0%)	0 (0%)	*p* = 0.041	*p* = 0.032

	GG	66 (88%)	56 (96.6%)	64 (98.5%)		
Prothrombin	GA	9 (12%)	2 (3.4%)	1 (1.5%)	3.8 (0.8–18.4)	4.3 (0.9–20.7)
G20210A	AA	0 (0%)	0 (0%)	0 (0%)	*p* = 0.095	*p* = 0.069

	GG	39 (52%)	33 (56.9%)	31 (47.7%)		
MTHFR	GA	25 (33.3%)	20 (34.5%)	30 (46.2%)	1.2 (0.6–2.4)	0.8 (0.4–1.6)
C677T	AA	11 (14.7%)	5 (8.6%)	4 (6.1%)	*p* = 0.57	*p* = 0.61

	GG	32 (42.7%)	25 (43.1%)	29 (44.6%)		
MTHFR	GA	33 (44%)	25 (43.1%)	31 (47.7%)	1.8 (0.6–5.6)	1.1 (0.6–2.1)
C1298A	AA	10 (13.3%)	8 (13.8%)	5 (7.7%)	*p* = 0.29	*p* = 0.81
